# Magnetic brightening and its dynamics of defect-localized exciton emission in monolayer two-dimensional semiconductor

**DOI:** 10.1126/sciadv.adr5562

**Published:** 2025-06-04

**Authors:** Yubei Xiang, Keisuke Shinokita, Kenji Watanabe, Takashi Taniguchi, Kazunari Matsuda

**Affiliations:** ^1^ Institute of Advanced Energy, Kyoto University, Uji, Kyoto 611-0011, Japan.; ^2^ Research Center for Electronic and Optical Materials, National Institute for Materials Science, 1-1 Namiki, Tsukuba, Ibaraki 305-0044, Japan.; ^3^ International Center for Materials Nanoarchitectonics, National Institute for Materials Science, 1-1 Namiki, Tsukuba, Ibaraki 305-0044, Japan.

## Abstract

Quantum light sources, especially single-photon emitters, are crucial for advancing quantum technologies. Despite extensive research, the behavior of defect-localized excitons in monolayer WSe_2_ under external perturbations, such as magnetic fields, remain underexplored. This study investigates the nature and dynamics of defect-localized excitons under in-plane magnetic fields using steady-state and time-resolved photoluminescence (PL) spectroscopy. Observations reveal a sharp PL peak, indicative of single-photon emission, with doublet peaks from hybridized spin–state excitons. Notably, magnetic brightening of the PL peak was detected at a low magnetic field (<1 tesla), and the dynamics of hybridized-state excitons under magnetic fields indicated field-induced state mixing, explaining the magnetic brightening. These findings advance tunable single-photon emitters controlled by magnetic fields, with implications for quantum optics applications.

## Teaser

The light emission with single-photon characteristics exhibits enhanced brightness under a magnetic field in monolayer WSe_2_.

## INTRODUCTION

Two-dimensional (2D) transition-metal dichalcogenides (TMDs), specifically MX_2_ compounds (M = Mo, W; X = S, Se, Te), have emerged as promising platforms for nanoscience and advanced optical devices due to unique electronic and optical properties, including layer thickness–dependent bandgaps (*1*–*4*). Monolayer semiconducting TMDs exhibit a direct bandgap at the K and K′ valleys in momentum space. The coupling of valley and spin degrees of freedom, or valley-spin locking, results in valley-dependent optical selection under circularly polarized light. Under this light, optically generated electron-hole pairs form valley excitons through strong Coulomb interactions and dielectric screening effects at the K (K′) valleys (*5*–*8*). Spin-dependent exciton transitions are classified as either optically allowed singlet bright or optically forbidden singlet dark (*9*, *10*). Experimental and theoretical studies have explored dark exciton control through defect engineering and external magnetic fields, both in-plane and out-of-plane (*11*–*17*). In addition, precise control over magnetic field strength and orientation enables manipulation of exciton spins, enhancing radiative recombination and brightening dark excitons (*18*).

Single-photon emitters are vital for quantum technologies, enabling advancements in quantum key distribution, quantum computing, and quantum sensing (*19*–*21*). Developing stable, efficient, and controllable single-photon sources is essential (*22*, *23*). Monolayer tungsten diselenide (WSe_2_) with a single defect shows promise as a single-photon source (*11*, *18*, *24*, *25*). Single-photon emission has been achieved from point defects, such as vacancies and antisites, introduced into monolayer WSe_2_ through annealing, plasma treatment, or ion irradiation (*26*–*28*). Defect-localized excitons feature two spin states, resulting from defect-induced mismatch and disorder, which disrupt spin-momentum locking in monolayer WSe_2_ (*29*–*31*). This spatial localization leads to the hybridization of bright and dark states, extending lifetimes (*14*, *30*, *32*). However, the dynamics of defect-localized excitons with two spin states remain unclear, and understanding the mixing and transition processes of these excitons under an external magnetic field is crucial for controlling single-photon emissions.

In this study, we examine the physical characteristics and dynamics of defect-localized excitons in monolayer WSe_2_ under a magnetic field. The sharp photoluminescence (PL) peak, indicative of single-photon emission, consists of doublet peaks with fine structure splitting, attributed to defect-localized exciton emissions originating from two hybridized spin states formed by a mixture of intra- and inter-valley bright and dark states due to valley symmetry breaking. In addition, we observed magnetic brightening of the PL peak even at relatively low magnetic fields (below 1 T). The dynamics of localized exciton states under magnetic fields, driving efficient magnetic brightening, are discussed on the basis of steady-state and time-resolved PL spectroscopy.

## RESULTS

### Defect-localized single-photon emitters

**Fig. 1.**
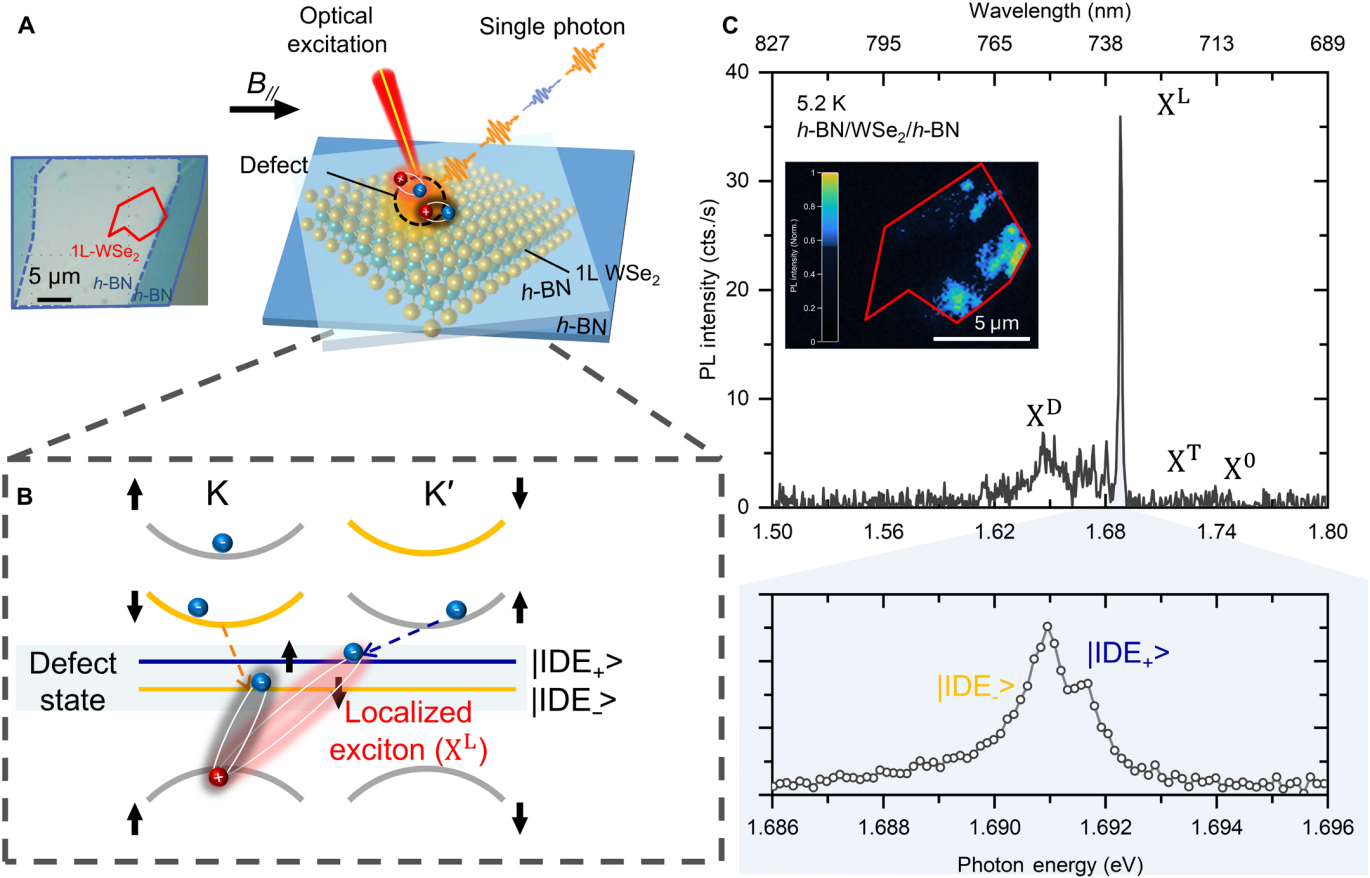
Defect-localized exciton emission from monolayer WSe_2_ encapsulated by *h-*BNs. (**A**) Schematic of 1 L-WSe_2_ with defect sites encapsulated by top and bottom *h*-BNs. Left corner, optical image of monolayer WSe_2_ encapsulated by *h-*BNs. The WSe_2_ monolayer and *h*-BN few layers are highlighted in red and blue lines, respectively. Scale bar, 5 μm. (**B**) Schematic of band structure and spin state in monolayer WSe_2_ with defect states in reciprocal space. Two-spin states formed by a point defect in the bandgap break the valley selectivity. The optical transition between the defect states corresponding to optically forbidden initially dark and optically allowed initially bright states is shown. (**C**) Top, low-temperature PL spectrum at 5.2 K under the excitation power of 100 nW. The inset shows the PL image of monolayer WSe_2_ encapsulated *h-*BNs with defect sites monitored below 1.77 eV at 5.2 K; the WSe_2_ monolayer is highlighted in red line. Bottom, low-temperature PL spectrum under excitation power of 100 nW with polarization-unresolved and high spectral resolution condition.

Figure 1A provides a comprehensive overview of this study. Monolayer WSe_2_ with emissive defects was carefully prepared through a controlled annealing process in vacuum, with pre-thermal treatment inherently introducing a substantial number of defects and strains that promoted defect emission (*33*, *34*). Monolayer WSe_2_, encapsulated within multilayered *h*-BNs, was transferred onto a Si/SiO_2_ substrate using the dry transfer method with a polymer stamp (see Materials and Methods). The inset of Fig. 1A shows an optical microscopy image of monolayer WSe_2_ encapsulated by *h*-BNs, with monolayer WSe_2_ and *h*-BNs outlined in red and blue, respectively.

Figure 1B illustrates a schematic of the band structure and spin states in monolayer WSe_2_ with defect states. The point defect forms in-gap states within the bandgap of monolayer WSe_2_, where optically excited excitons are trapped and localized at defect sites. This defect disrupts valley symmetry, creating a mixture of intra- and intervalley optically allowed bright and optically forbidden dark excitons at the intervalley defect excitonic (IDE) states, resulting in doublets |IDE_+_> and |IDE_−_> with different spin states and a small splitting energy δ due to fine structure splitting, giving rise to intervalley defect emissions (*18*, *30*, *35*, *36*).The top of Fig. 1C shows the PL spectrum of monolayer WSe_2_ at 5.2 K under an excitation power of 100 nW. Several sharp peaks are clearly visible in the spectrum. The peaks at 1.71 and 1.73 eV correspond to charged exciton or trion (X^T^) and free exciton (X^0^) (*37*). The sharp, strong peaks ranging between 1.55 and 1.70 eV are attributed to defect-related exciton emissions (X^D^) from the narrow linewidth, energy position, and intensity dependent on defect type and quantity as reported in previous studies (*38*–*40*). In addition, the sharpest and strongest peak was identified as a defect-localized single-photon emitter (XL). The photon energy of the defect-localized emission peak varies by sample and position (see fig. S1). The sharp peak comprises two closely spaced doublet peaks with small energy splitting (<1 meV) (*18*, *30*, *31*). The lower part of Fig. 1C shows an expanded PL spectrum measured at higher resolution, displaying doublet peaks with less than 1 meV splitting between 1.686 and 1.696 eV, which will be discussed further. The PL image at 5.2 K is shown in the inset of Fig. 1C, monitored below 1.77 eV, revealing several bright and isolated spots that indicate spatially localized defect-related exciton emissions.

**Fig. 2.**
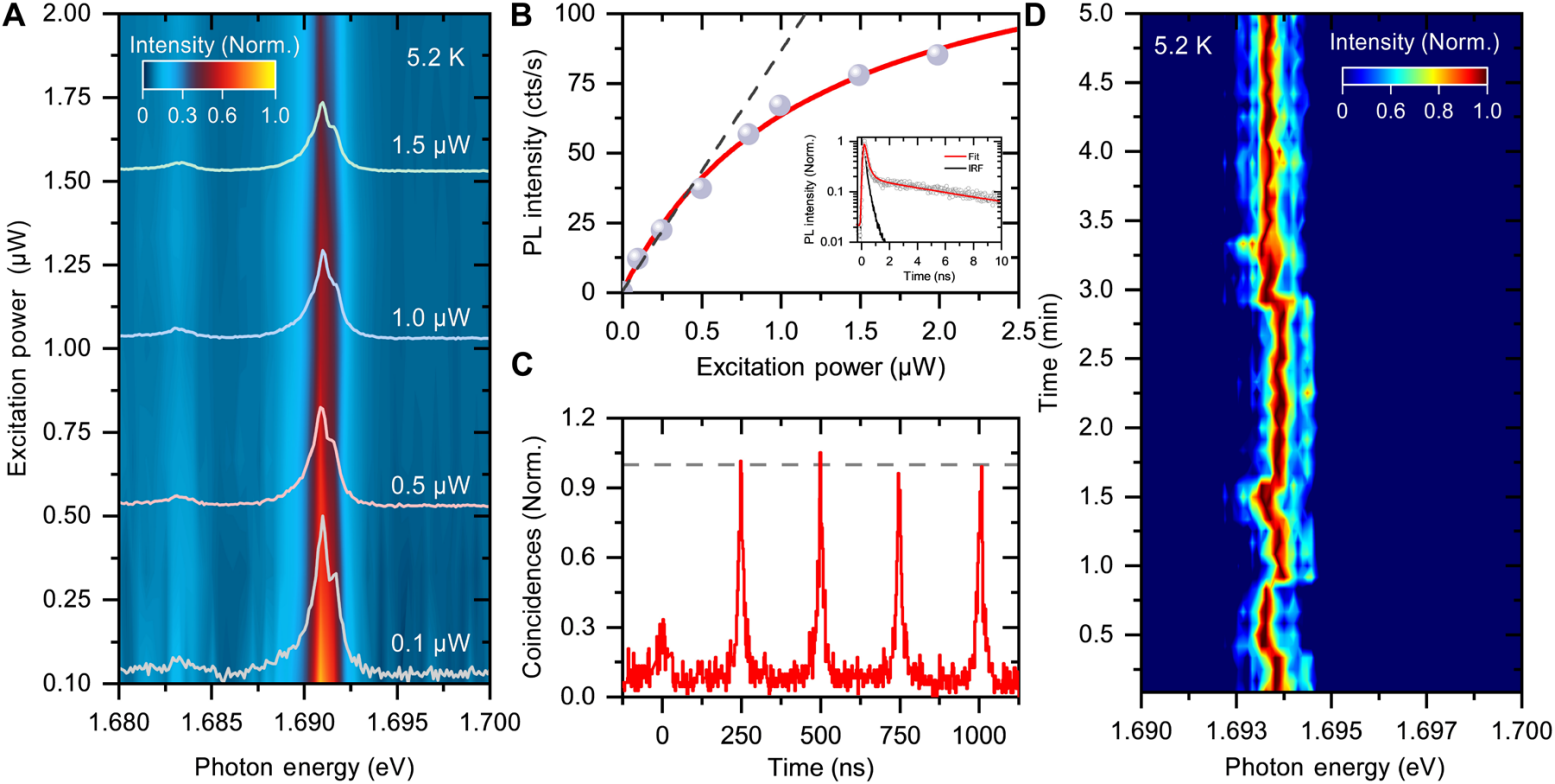
Defect-localized single photon emission from monolayer WSe_2_ encapsulated by *h-*BNs. (**A**) PL spectra and contour plot of low-temperature PL spectra of monolayer WSe_2_ encapsulated by *h-*BNs with defect sites under excitation power conditions from 0.1 to 2.0 μW. (**B**) Integrated PL intensities as a function of excitation power. The right inset, the time-resolved PL decay profiles monitored at the photon energy of 1.69 eV. (**C**) Second-order photon correlation *g*^2^(τ) from localized exciton emission (sample 2), where the cross-talk signals in the histogram are removed. (**D**) Contour plot of time evolution of PL spectrum from defect site emission at low temperature at 5.2 K under the excitation power of 1 μW. Accumulation time for each spectrum is 5 s.

The PL spectra were measured at a low temperature (5.2 K) with increasing excitation power. Figure 2A shows the PL spectra and a contour map of power-dependent spectra from 100 nW to 2 μW, where the PL intensity in the contour map is normalized by the excitation power. The integrated PL intensity as a function of excitation power is plotted in Fig. 2B. The integrated PL intensity increases linearly under weak excitation conditions below 0.4 μW, as indicated by a gray dashed line for guidance. As the excitation power increased, the PL intensity gradually approached saturation at higher excitation powers. The excitation power dependence of the PL intensity was fitted using a phenomenological model for saturation behavior, represented by the solid red line (*41*)

$$I=\frac{I_{\text{sat}}\cdot P}{P_{\text{sat}}+P}$$ (1)

where *I*_sat_ and *P*_sat_ denote the saturated PL intensity and power of emission, respectively. The experimental results of the excitation power dependence of the integrated PL intensity align with the phenomenological model, yielding a saturation power of 1.2 μW. The right inset in Fig. 2B shows time-resolved PL decay profiles fitted by a double-exponential function, from which an effective lifetime of 9.7 ns was obtained, to be discussed later. In addition, the optically generated exciton density under steady-state conditions can be calculated as follows

$$g_{\text{eff}}=\tau_{\text{eff}}\times \frac{\left(1-R\right)\alpha \mathrm{dP}}{\hbar \omega }$$ (2)

where the τ_eff_ is the effective lifetime, *R* represents the reflectivity (=0.4), α is the absorption coefficient (2 × 10^5^ cm^−1^), *d* stands for the monolayer thickness (0.7 nm), *P* is the power density (≈63 Wcm^−2^ at a laser spot size of 1.4 μm in diameter), and ℏω refers to the photon energy of 1.95 eV. This calculation yields an exciton density of 3.4 × 10^10^ cm^−2^ under saturation conditions, which is relatively low compared to the exciton density of 2D free excitons in monolayer semiconductors (*37*, *42*).

Figure 2C shows photon statistics for the sharp spectral peak at 1.629 eV observed in a different monolayer of WSe_2_ (sample 2; see fig. S1A), encapsulated within multilayered *h*-BNs on a Si/SiO_2_ substrate and measured using a Hanbury-Brown and Twiss (HBT) setup (see Materials and Methods) under 4-MHz pulsed laser excitation. The normalized second-order correlation function of emitted photons at zero delay time $g^{2}\left(0\right)$ shows a clear decrease compared to the values at each 250-ns interval. The decrease of $g^{2}\left(0\right)$ to approximately 0.3, notably below the 0.5 threshold, reveals the antibunching characteristic of photon emission from a single quantum state (*43*, *44*). This result confirms that defect-localized exciton emission functions as a single-photon emitter.

Figure 2D shows a contour plot of the time evolution of the PL spectrum measured at a low temperature (5.2 K) under an excitation power of 1 μW, with an accumulation time of 5 s for each spectrum. Spectral wandering on an energy scale of about 600 μeV results in inhomogeneous broadening of the spectral peaks. The peak energy splitting and intensity ratio of the doublet peaks remained nearly constant, suggesting that the doublet peaks originate from two states within a single defect in monolayer WSe_2_ (also see fig. S2, A and B) (*40*, *44*–*46*).

### Defect-localized exciton dynamics

**Fig. 3.**
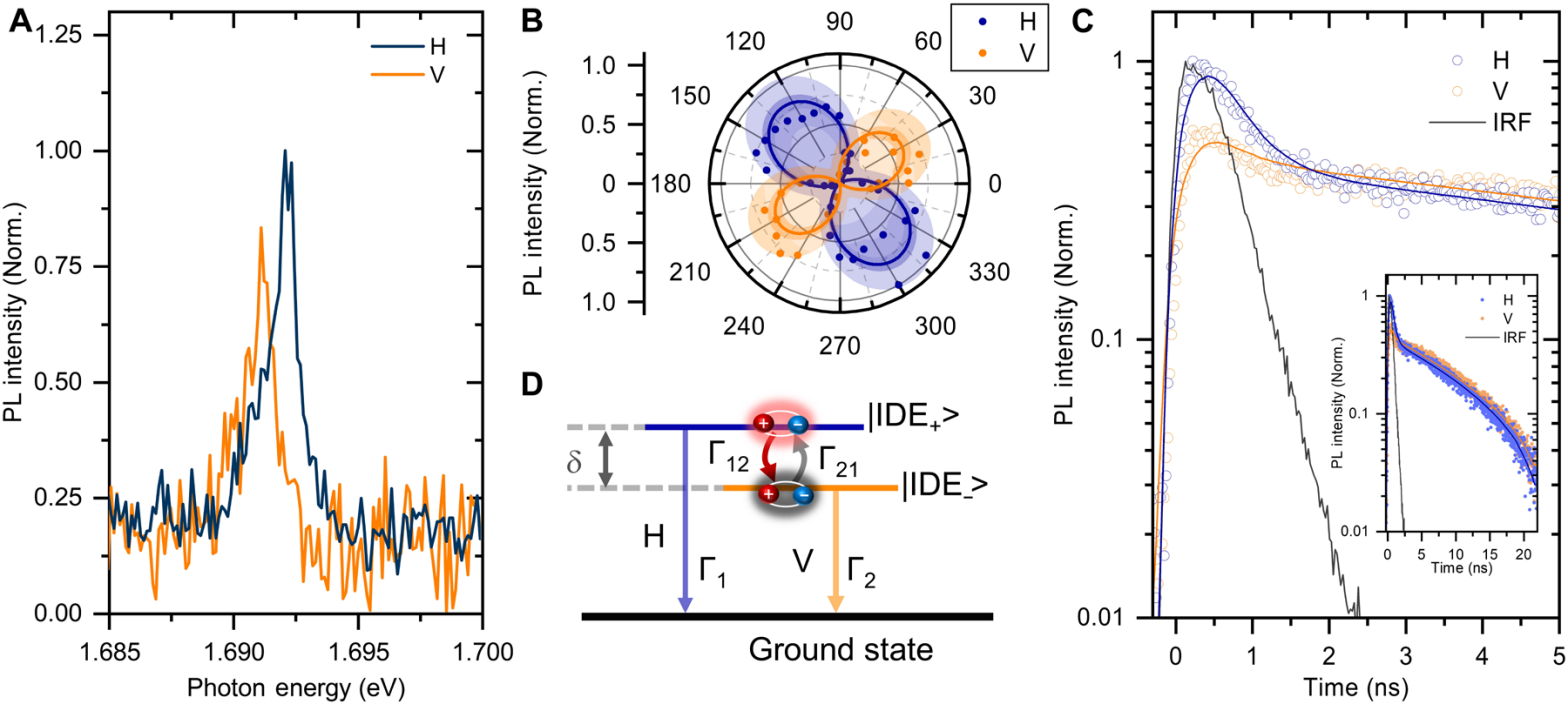
Polarization and time-resolved PL spectroscopy from defect site emission at low temperature. (**A**) Polarization-resolved PL spectra with horizontal (H) and vertical (V) linearly polarized configuration. (**B**) Polar plot of integrated PL intensity of H and V detected configuration, where the angle value is the absolute orientation of the detection in the experimental conditions. (**C**) Time-resolved PL decay profiles detected in H and V configurations monitored at the photon energy of 1.69 eV on the initial range of 5-ns windows. Inset, time-resolved PL spectra with H and V configuration in the range of 20-ns windows. (**D**) Schematic of the three-level system used in the rate equation analysis for defect localized exciton with hybridized exciton states and ground state.

Figure 3A shows the polarization-resolved PL spectra obtained in the polarization direction in the horizontal (H) and vertical (V) configurations, and are plotted as blue and orange solid lines, respectively. The doublet PL spectra were separated into the higher-energy and lower-energy peaks in the H- and V-polarization configurations. The PL intensity and peak position varied strongly depending on the detected polarization angle, suggesting that the doublets |IDE_+_> and |IDE_−_> with different spin states and small splitting energies exhibited different polarization dependences originating from the two hybridized intra- and intervalley bright and dark excitons trapped and hybridized at the defect site (*30*, *31*). A detailed analysis of the sharp PL peak was fitted by the sum of Voigt functions with a photon energy difference within 1 meV. Figure 3B shows the polar plot of the integrated PL intensity as a function of the detected linear polarization angle. The solid line shows a sinusoidal fit of the results, and the dense and light-shaded areas show the 95% confidence and prediction limits on the upper and lower sides, respectively. The experimental results of the polar plot clearly show that each doublet peak has a twofold symmetric and orthogonal pattern. Moreover, the merged doublet peaks from |IDE_+_> and |IDE_−_> in the bottom of Fig. 1C can be clearly resolved by selecting the linear polarization directions detected in the H and V configurations.

Time-resolved PL decay profiles (See Materials and Methods) were measured at sharp spectral peaks obtained in the polarization direction of H and V configurations. Figure 3C shows the PL decay profiles in the initial range of decay in a 5-ns window, while the right inset shows the time-resolved PL decay profiles in the range of 20-ns windows, monitored at the H (blue circle) and V (orange circle) configurations, where the intensity is normalized by the total PL intensity from both polarization direction configurations. Two decay components with lifetimes of a few nanoseconds and several tens of nanoseconds are clearly observed. The PL decay profile was modeled using double-exponential decay functions (*47*) as follows

$$I\left(t\right)=A_{f}\text{exp}\left(-\frac{t}{\tau_{f}}\right)+A_{s}\text{exp}\left(-\frac{t}{\tau_{s}}\right)$$ (3)

where the *A*_f_ and *A*_s_ are the amplitudes of the fast and slow components, and $\tau_{f}^{-1}$ and $\tau_{s}^{-1}$ represent the fast and slow decay rates, respectively. Moreover, the effective lifetime is defined as $\tau_{\text{eff}}=\frac{A_{f}\tau_{f}+A_{s}\tau_{s}}{A_{f}+A_{s}}$ (*47*, *48*).

The decay profiles were calculated using Eq. 3, considering the convolution procedure of the instrumental response function as the gray line, reproduces the characteristic PL decay profile well. The fitted results for different polarization directions in the H and V directions are plotted as blue and orange curves in Fig. 3C, which accurately reproduces the experimental results. The parameters of amplitudes of *A*_f(s)_ and decay rates of $\tau_{f\left(s\right)}^{-1}$ for H and V configuration are obtained, which will be used for later analysis based on the rate equations. Moreover, the integrated intensity ratio of the H and V configurations from the time-resolved PL decay profiles corresponded well to the doublet PL peaks of the higher- and lower-energy sides at about 1.692 and 1.691 eV, respectively.

From the time-resolved PL decay profiles within the initial 5-ns window, the amplitude of the fast decay component in the H configuration is greater than in the V configuration, suggesting a higher fast-to-slow amplitude ratio in the H configuration. To understand this, we analyzed the PL decay dynamics of defect-localized excitons using phenomenological models based on the coupled rate equations. A three-level model was considered (*42*, *47*–*49*), as illustrated in Fig. 3D, consisting of defect-localized excitons with hybridized states and crystal ground states. The point defect in the monolayer WSe_2_ breaks the valley symmetry, mixing intra- and intervalley bright and dark excitons at the defect level. This mixing leads to the formation of doublets, |IDE_+_> and |IDE_−_> with different spin states and a small splitting energy (δ). The time-dependent populations of excitons in |IDE_+_> and |IDE_−_>, represented by *N*_1_(t) and *N*_2_(t), are as follows

$$\frac{dN_{0}\left(t\right)}{\mathrm{dt}}=-G\times N_{0}\left(t\right)\times \left(p_{1}+p_{2}\right)+\Gamma_{1}\times N_{1}\left(t\right)+\Gamma_{2}\times N_{2}\left(t\right)$$ (4)

$$\begin{array}{c} \frac{dN_{1}\left(t\right)}{\mathrm{dt}}=G\times N_{0}\left(t\right)\times p_{1}\times \left[1-\frac{N_{1}\left(t\right)}{n_{1}}\right]-\\ \left\{\Gamma_{1}+\Gamma_{12}\times \left[1-\frac{N_{2}\left(t\right)}{n_{2}}\right]\right\}\times N_{1}\left(t\right)+\Gamma_{21}\times \left[1-\frac{N_{1}\left(t\right)}{n_{1}}\right]\times N_{2}\left(t\right) \end{array}$$ (5)

$$\begin{array}{c} \frac{dN_{2}\left(t\right)}{\mathrm{dt}}=G\times N_{0}\left(t\right)\times p_{2}\times \left[1-\frac{N_{2}\left(t\right)}{n_{2}}\right]-\\ \left\{\Gamma_{2}+\Gamma_{21}\times \left[1-\frac{N_{1}\left(t\right)}{n_{1}}\right]\right\}\times N_{2}\left(t\right)+\Gamma_{12}\times \left[1-\frac{N_{2}\left(t\right)}{n_{2}}\right]\times N_{1}\left(t\right) \end{array}$$ (6)

where *G* represents the total generation rate of the defect-localized exciton, and *p*_1_ and *p*_2_ are the occupation probabilities of |IDE_+_> and |IDE_−_>, respectively. The variables $N_{i}$ and $n_{i}$ (*i* = 0, 1, 2) indicate the occupation number and maximum allowed excitons for each state. The transitions between |IDE_+_> and |IDE_−_> are represented by Γ_12_ and Γ_21_, while transitions from |IDE_+_> and |IDE_−_> to the ground state are denoted by Γ_1_ and Γ_2_, respectively. The transition rates Γ_12_ and Γ_21_ can be expressed as, $\Gamma_{12}=\gamma_{0}\left(n+1\right)$ , and $\Gamma_{21}=\gamma_{0}n$ , using the phonon occupation number n $\left(={\left\{\text{exp}\left(\delta /k_{B}T\right)-1\right\}}^{-1}\right)$ , where $k_{B}$ is the Boltzmann constant, *T* is the temperature, δ is the energy splitting between |IDE_+_> and |IDE_−_> states, and γ_0_ is the temperature-independent scattering rate (*47*, *49*).

The solutions for *N*_1_(*t*) and *N*_2_(*t*) can be determined by solving the coupled rate equations under steady-state conditions (see Materials and Methods) and the initial populations of |IDE_+_> and |IDE_−_>, $N_{1}\left(0\right):N_{2}\left(0\right)=p_{1}:p_{2}$ , $N_{1}\left(0\right)+N_{2}\left(0\right)=1$ , and $N_{0}\left(0\right)=0$ (see note S1). The obtained result can be simplified to a bi-exponential function as follows

$$N_{1\left(2\right)}\left(t\right)=A_{f,1\left(2\right)}\text{exp}\left(-\frac{t}{\tau_{f}}\right)+A_{s,1\left(2\right)}\text{exp}\left(-\frac{t}{\tau_{s}}\right)$$ (7)

The fast and slow decay rates ${\tau_{f}}^{-1}$ and ${\tau_{s}}^{-1}$ are described as follows

$${\tau_{f\left(s\right)}}^{-1}=\frac{1}{2}\left(\Gamma_{1}+\Gamma_{2}+\Gamma_{12}+\Gamma_{21}\pm H\right)$$ (8)

where *H* is the difference between the fast and slow decay rates and can be described as

$$H=\sqrt{{\left(\Gamma_{1}-\Gamma_{2}+\Gamma_{12}-\Gamma_{21}\right)}^{2}+4\Gamma_{12}\Gamma_{21}}$$ (9)

We further determine the amplitude factors [*A*_f,1(2)_ and *A*_s,1(2)_] for *N*_1_(*t*) and *N*_2_(*t*) as follows

$$A_{f\left(s\right),1}=\mp N_{1}\left(0\right)\frac{\Gamma_{2}+\Gamma_{21}-{\tau_{f\left(s\right)}}^{-1}}{{\tau_{f}}^{-1}-{\tau_{s}}^{-1}}\pm N_{2}\left(0\right)\frac{\Gamma_{21}}{{\tau_{f}}^{-1}-{\tau_{s}}^{-1}}$$ (10)

$$A_{f\left(s\right),2}=\mp N_{1}\left(0\right)\frac{\Gamma_{12}}{{\tau_{f}}^{-1}-{\tau_{s}}^{-1}}\mp N_{2}\left(0\right)\frac{\Gamma_{1}+\Gamma_{12}-{\tau_{f\left(s\right)}}^{-1}}{{\tau_{f}}^{-1}-{\tau_{s}}^{-1}}$$ (11)

We derived the obtained transition rates of Γ_12(21)_ and Γ_1(2)_ from the experimentally obtained parameters of *A*_f(s)_ and decay rates of $\tau_{f\left(s\right)}^{-1}$ for H and V configurations using Eqs. 7 to 11. Herein, the initial population ratio of excitons in |IDE_+(−)_> denoted as *N*_1(2)_(0) is assumed as 0.62:0.38 (see note S1).

On the basis of the above rate equation analysis, the PL intensities of the |IDE_+_> and |IDE_−_> states, $I_{|{\text{IDE}}_{+}>}$ and $I_{|{\text{IDE}}_{-}>}$can be expressed under steady-state conditions (see note S1) using Eq. 12

$$\begin{array}{c} \begin{array}{c} I_{|{\text{IDE}}_{+}>\left(|{\text{IDE}}_{-}>\right)}\propto \Gamma_{1\left(2\right)}\cdot \\ \frac{G\times \left[\Gamma_{2\left(1\right)}N_{1\left(2\right)}\left(0\right)+\Gamma_{21\left(12\right)}\right]}{G\times \left[\Gamma_{2}N_{1}\left(0\right)+\Gamma_{21}+\Gamma_{1}N_{2}\left(0\right)+\Gamma_{12}\right]+\Gamma_{1}\Gamma_{21}+\Gamma_{2}\Gamma_{12}+\Gamma_{1}\Gamma_{2}} \end{array} \end{array}$$ (12)

The PL intensity ratio of $I_{|{\text{IDE}}_{-}>}$ and $I_{|{\text{IDE}}_{+}>}$ defined as $I_{R}\left(\equiv I_{|{\text{IDE}}_{-}>}/I_{|{\text{IDE}}_{+}>}\right)$ , which reflects the hybridization of two states is described using Eq. 13,

$$I_{R}\equiv I_{|{\text{IDE}}_{-}>}/I_{|{\text{IDE}}_{+}>}=\frac{\Gamma_{2}\times \left[\Gamma_{1}N_{2}\left(0\right)+\Gamma_{12}\right]}{\Gamma_{1}\times \left[\Gamma_{2}N_{1}\left(0\right)+\Gamma_{21}\right]}$$ (13)

### Magnetic field–dependence exciton dynamics

**Fig. 4.**
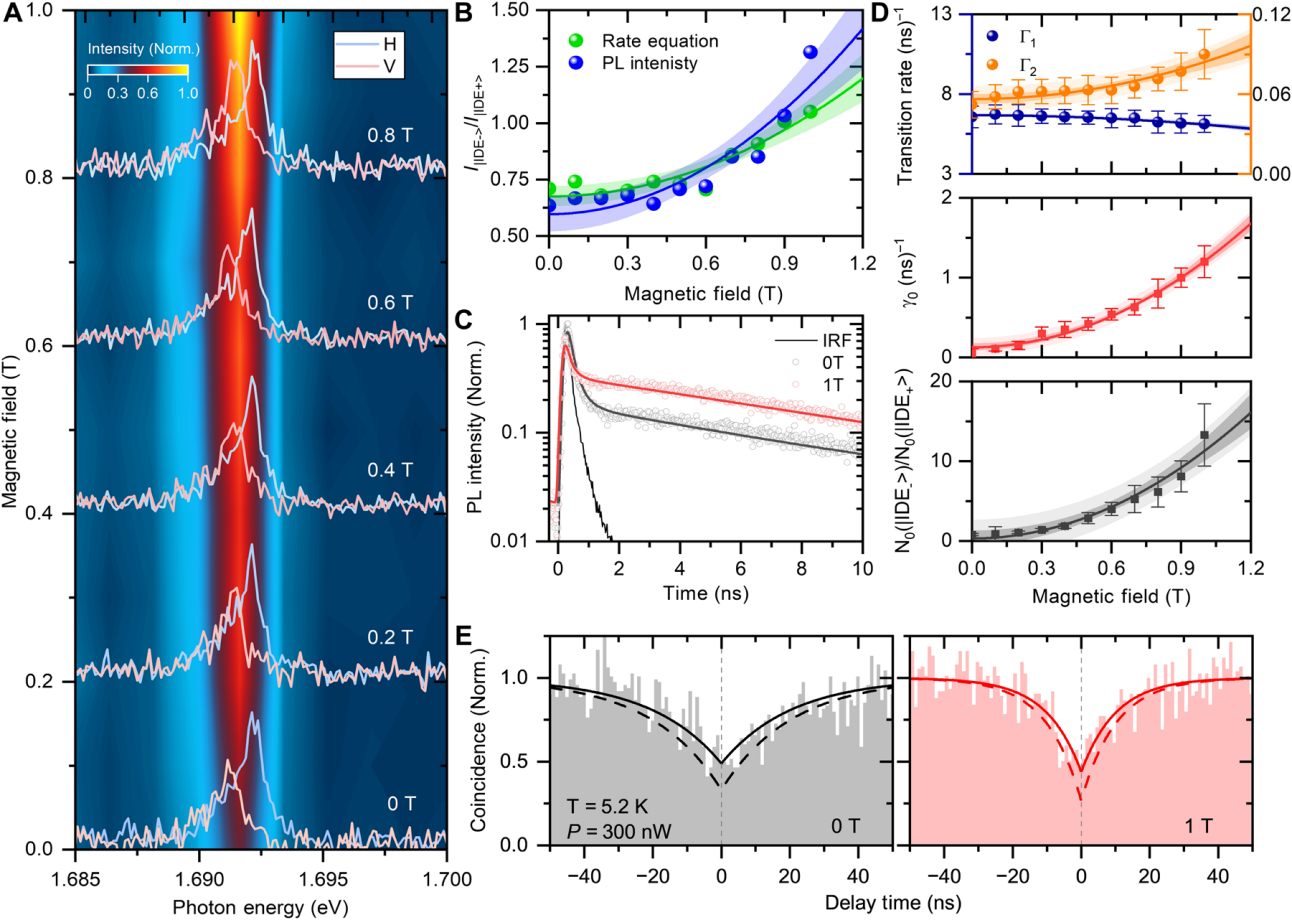
Magnetic field dependent PL spectroscopy from defect site emission at low temperature. (**A**) Contour plot of low-temperature PL spectra of monolayer WSe_2_ encapsulated *h-*BNs with defect sites with applying in-plane magnetic field from 0 to 1 T under excitation power of 250 nW at 5.2 K with polarization-unresolved, and low spectral resolution condition. The solid line shows polarization-resolved PL spectra with high spectral resolution condition in each magnetic field. (**B**) Experimental results of polarization-resolved PL intensity ratio of doublet peaks from |IDE_−_> (V) and |IDE_+_> (H) (blue) and analysis results from the rate equation (green). (**C**) Time-resolved PL decay profiles monitored at the photon energy of 1.69 eV at 0 and 1 T. (**D**) Evaluation results from the rate equation analysis: Top, magnetic field dependence of transition rates Γ_1_ (blue), and Γ_2_ (orange) from |IDE_+_> and |IDE_−_> to the ground states. Middle, temperature independent scattering rate γ_0_ between |IDE_+_> and |IDE_−_>. Bottom, ratio of initial exciton populations in |IDE_−_> and |IDE_+_> states evaluated from the rate equation analysis. The error bars show the range of solutions derived from the rate equation that agree with the experimental obtained parameters within the accuracy of 90%. (**E**) Experimental results of the photon correlation as a function of the delay time from −50 to 50 ns from localized exciton emission (sample 4) using the continuous-wave laser of 300 nW at 5.2 K at 0 (left, black) and 1 T (right, red). The solid and dashed lines correspond to a fitting curve of $C_{N}\left(\tau \right)$ and calibrated $g^{2}\left(\tau \right)$ considering the imbalanced noise caused by dark counts.

The dynamics of localized hybridized excitons were investigated through magnetic-field-dependent steady-state and time-resolved PL spectroscopy in Voigt geometry. Figure 4A shows a contour map of the magnetic field–dependent PL spectra from 0 to 1 T under an excitation power of 250 nW at 5.2 K. A series of PL spectra are presented in fig. S3A. Notable changes in PL intensity with an increasing magnetic field are evident in the contour map in Fig. 4A (see also fig. S3A). The polarization-resolved PL spectra under various magnetic fields are shown as solid lines in Fig. 4A, with each spectrum normalized to the peak intensity of the higher-energy states for each magnetic field. The PL intensity of excitons in the |IDE_−_>states on the lower-energy side gradually increases with the external magnetic field, suggesting magnetic brightening of the |IDE_−_> excitons. The PL intensity ratio $I_{R}\left(=I_{|{\text{IDE}}_{-}>}/I_{|{\text{IDE}}_{+}>}\right)$ of |IDE_−_> and |IDE_+_> states as a function of the magnetic field is plotted in Fig. 4B. The PL intensity ratio $I_{R}$ clearly displays a characteristic quadratic increase with the magnetic field B, as $I_{R}$ = α*B*^2^, where α is the magnetic brightening factor. This behavior is similar to the magnetic brightening of dark excitons in monolayer semiconductors and carbon nanotubes (*11*–*13*, *50*, *51*). Notably, the magnetic brightening factor for the hybridized localized excitons trapped in monolayer WSe_2_ defects (α ~ 0.57) is much higher than that observed in dark excitons in monolayer semiconductors and carbon nanotubes (see table S1), indicating that magnetic brightening is detectable even under a relatively small magnetic field below 1 T.

Magnetic-induced mixing occurs between the |IDE_+_> (bright) and |IDE_−_> (dark) doublet states. The oscillator strength ratio between the dark |IDE_−_> state and the bright |IDE_+_> state can be predicted as $f_{D}/f_{B}={\left(\mu_{B}B_{∥}/∆E\right)}^{2}$ , where *f*_D_ and *f*_B_ is the oscillator strength of the dark |IDE_−_> and bright |IDE_+_ > excitons, respectively, $B_{∥}$ is the external magnetic field, ∆E is the energy splitting between these exciton states, and μ_Β_ is the Bohr magneton (*12*, *13*, *50*). Even a small magnetic field on the order of 1 T leads to a notable enhancement in the oscillator strength of the dark |IDE_−_> state due to wave function mixing in the very small energy splitting between the two states ( ∆E< 1 meV). Moreover, the external magnetic field influences the dynamics of the dark |IDE_−_> state, affecting radiative processes and scattering between the bright and dark states. The transition probability between the bright and dark states, determined by the scattering rate $\gamma_{0}$ will be accelerated, which also affects the efficient magnetic brightening phenomenon of defect-localized exciton emission.

We further analyzed the time-resolved PL decay results with an external magnetic field. Figure 4C compares the PL decay profiles measured at 0 and 1 T under an excitation power of 250 nW at 5.2 K (see also fig. S3B). The relative amplitude of the slow decay component primarily reflects the increase in exciton emission in the lower-energy |IDE_−_> state, consistent with the experimental results that show an increase in the relative PL intensity in the |IDE_−_> exciton state. The PL decay profiles measured at 0 and 1 T were fitted with double-exponential functions using Eq. 3, as indicated by the solid lines in Fig. 4C, accurately reproducing the experimental results. The parameters of the decay rates [ $\tau_{f\left(s\right)}^{-1}$ ] as a function of the external magnetic field were obtained using a double-exponential function (red) using Eq. 3 and the rate equation analysis (blue) (refer to note S1), which align well, as shown in fig. S3 (C and D). From these parameters, the decay rate of Γ_1(2)_ in |IDE_+_> and |IDE_−_>, the transition rate of Γ_12(21)_ between |IDE_+_> and |IDE_−_>, and the initial population *N*_1(2)_(0) in |IDE_+_> and |IDE_−_> were derived as functions of external magnetic fields.

The top of Fig. 4D shows the evaluated decay rate of Γ_1(2)_. The decay rate of Γ_1_ in the bright |IDE_+_> (blue) exciton decreases, while the decay rate of Γ_2_ in the dark |IDE_−_> (orange) increases. The temperature-independent scattering rate γ_0_ between |IDE_+_> and |IDE_−_> at each magnetic field *B* is shown in the middle of Fig. 4D. The scattering rate γ_0_ shows a sharp increase with the magnetic field *B*. In the absence of an external magnetic field, the bright |IDE_+_> exciton undergoes rapid relaxation, while the dark |IDE_−_> exciton has a longer lifetime, governed by the radiative decay rate of Γ_1_ and Γ_2_. However, when the in-plane magnetic field breaks the spin-momentum symmetry between the |IDE_+_> and |IDE_−_> states, phonon-assisted scattering between |IDE_+_> and |IDE_−_> occurs. Even in relatively low in-plane magnetic fields below 1 T, the scattering between |IDE_+_> and |IDE_−_> increases substantially. The ratio of the initial exciton populations in the |IDE_−_> and |IDE_+_> states [ $N_{2}\left(0\right)$/$\;N_{1}\left(0\right)]$ evaluated from the rate equation analysis, is shown in the bottom of Fig. 4D. The initial exciton population in the dark |IDE_−_> state increases with the external magnetic field due to field-induced mixing between the two states. This analysis’s validity is supported by the calculated magnetic field dependence of the increase in the PL intensity ratio $I_{R}\left(B\right)\left[\equiv I_{|{\text{IDE}}_{-}>}\left(B\right)/I_{|{\text{IDE}}_{+}>}\left(B\right)\right]$ using Eq. 13, which matches well with the experimental results shown in Fig. 4B. Analysis based on the rate equation model suggests that defect-related single-photon emitters in monolayer WSe_2_ originate from defect-localized excitons in the two-spin states |IDE_+_> and |IDE_−_>. The external magnetic field breaks symmetry and induces a mix of |IDE_+_> and |IDE_−_> states, resulting in the magnetic brightening of the defect-localized emission. We also confirmed that our experimental findings of magnetic brightening and the change of PL decay dynamics under magnetic field are commonly verified across the samples and positions (see fig. S4).

The photon statistics from the photon correlation measurement further supports this dynamics analysis of the defect-localized single-photon emission with an external magnetic field, which simultaneously reveals the single-photon characteristic and the decay dynamics towards the stationary regime. The second-order correlation function $g^{2}\left(\tau \right)$ is conducted experimentally in HBT configuration under magnetic field using a fabricated sample (sample 4; see Materials and Methods and fig. S5), which is designed to improve collection efficiency of the emitted photons (*52*). Figure 4E shows the photon correlation measurements using the continuous-wave laser of 300 nW at 5.2 K (see also fig. S5D). The black and red curves displayed in the left and right parts represent the second-order correlation $g^{2}\left(\tau \right)$ of the defect-localized emission as a function of delay time at 0 and 1 T, respectively. The solid line represents a fitting curve using $C_{N}\left(\tau \right)=1-\left(1+\beta \right)\text{exp}\left(-\frac{|\tau |}{\tau_{A}}\right)+\beta \;\text{exp}\left(-\frac{|\tau |}{\tau_{B}}\right)$ , where τ is the time delay between two consecutive detected photons, β is the amplitude of the bunching and antibunching components, and $\tau_{A\left(B\right)}^{-1}$ represent the decay rates (*53*). The dashed line represents the calibrated $g^{2}\left(\tau \right)$ , which accounts for the imbalanced noise caused by dark counts (see note S2) (*52*, *54*). The second-order correlation function near zero delay time, *g*^2^(0), exhibits a clear symmetric decrease compared to the value at nonzero delay times, indicating the antibunching characteristic of single-photon emission. Specifically, $g^{2}\left(\tau \right)$ drops to approximately 0.35 at 0 and 0.27 at 1 T, both of which are below the value of 0.5. This result confirms the preserved antibunching characteristic, suggesting that the single-photon purity is maintained under the magnetic field. In addition, the decrease near zero delay time occurs more rapidly under the magnetic field of 1 T, and the characteristic dip at zero delay time becomes sharper in contrast to the case without the magnetic field, reflecting the accelerated decay dynamics of defect-localized single-photon emission under the magnetic field.

The photon correlation measurements using the pulsed laser excitation align well with the results using the continuous-wave excitation conditions (see fig. S5E). The normalized second-order correlation functions of the emitted photons at zero delay time, $g^{2}\left(0\right)$ , decrease to approximately 0.2 at both 0 and 1 T. The curve at every period interval becomes sharper under the magnetic field of 1 T compared to the case without the external magnetic field, and the coincidence counts at the overlap regions between consecutive periods show a notable decrease under the magnetic field. The changes of $g^{2}\left(\tau \right)$ indicate a reduction in the effective lifetime of the single-photon emission, resulting in the magnetic brightening of the defect-localized emission. Moreover, the calculated results of second-order correlation function $g^{2}\left(\tau \right)$ based on the rate equation and the simulations of sample 1 are well-consistent with the experimental results of sample 4 (see note S2 and fig. S6). The results of calculated photon correlation and their dynamics confirm single-photon purity under a magnetic field and reflect the magnetic field–induced dynamics of defect-localized excitons in monolayer WSe_2_.

## DISCUSSION

We investigated the nature and dynamics of defect-localized excitons in monolayer WSe_2_ under a magnetic field. A sharp PL peak, attributed to defect-localized emission, exhibited photon antibunching behavior in second-order correlation function measurements, indicating that it acts as a single-photon emitter. This sharp PL peak, comprising doublet peaks with fine-structure splitting of less than 1 meV, originates from defect-localized exciton emissions of two hybridized intra- and intervalley bright and dark excitons trapped and hybridized at the defect site. In addition, magnetic brightening of the |IDE_−_>exciton state occurs even in relatively low magnetic fields below 1 T, which is much weaker than similar effects in other spin-related free dark excitons in monolayer semiconductors and carbon nanotubes. Furthermore, the dynamics of localized excitons in the |IDE_+_> and |IDE_−_> states under magnetic fields demonstrate field-induced mixing, driving the magnetic brightening phenomena of defect-localized exciton emission. Our findings on the microscopic physical properties and dynamics of defect-localized exciton states illuminate magnetic brightening and suggest a new strategy for single-photon emitters controllable by an external magnetic field for quantum optics applications.

## MATERIALS AND METHODS

### Sample preparation

Monolayer WSe_2_ and a few layers of *h*-BNs were prepared by mechanical exfoliation from single crystals. The monolayer WSe_2_ was annealed at 150°C under vacuum conditions for 30 min to introduce intrinsic defects and relieve strain. The annealed monolayer WSe_2_ was encapsulated with top and bottom *h*-BNs. The sample was designed to be placed on either a Si/SiO_2_ substrate (sample 1, 2, and 3) or a planar Au substrate (sample 4, which enhances the collection efficiency of emitted photons). The encapsulation and transfer were performed using the dry transfer method with a poly(methyl 2-methylpropenoate) stamp. Unless otherwise specified, results are from sample 1.

### Steady-state PL measurements

A linearly polarized semiconductor laser (1.95 eV) served as the excitation light source for excitation power dependence and polarization-resolved PL spectra measurements. The laser was focused onto the sample surface with a 50× objective lens with a numerical aperture of 0.67, and an optical image was acquired. Samples were placed in a cryogen-free cryostat with a temperature range of 5.2 to 300 K. PL spectra were recorded using a monochromator equipped with a charge-coupled device camera.

### Time-resolved and second-order correlation PL measurements

An energy-filtered pulsed supercontinuum light source with a photon energy of 1.79 eV was used for time-resolved PL (pulse width ~20 ps; repetition rate 40 MHz) and second-order photon correlation measurements (pulse width ~100 ps; repetition rate 4 MHz or 10 MHz). A band-pass filter with an energy range of 1.687 to 1.710 eV captured the decay signal from the spectrum. Photon correlation measurements were performed in HBT configurations using Si avalanche photodiodes.

## Supplementary Materials

### This PDF file includes:

Supplementary Notes S1 and S2 Figs. S1 to S6 Table S1
