# Supplementary material for: Magnetic brightening and its dynamics of defect-localized exciton emission in monolayer two-dimensional semiconductor
Source: Sci Adv. 2025 Jun 4;11(23):eadr5562. doi: 10.1126/sciadv.adr5562 (PMC12136043; doi:10.1126/sciadv.adr5562)
Supplement: Supplementary file 1 — Supplementary Notes S1 and S2 Figs. S1 to S6 Table S1 [file sciadv.adr5562_sm.pdf]

Science Advances
AAAS

Supplementary Materials for

Magnetic brightening and its dynamics of defect-localized exciton emission in monolayer two-dimensional semiconductor

Yubei Xiang *et al.*

Corresponding author: Kazunari Matsuda, matsuda@iae.kyoto-u.ac.jp

Sci. Adv. **11**, eadr5562 (2025)
DOI: 10.1126/sciadv.adr5562

This PDF file includes:

Supplementary Notes S1 and S2
Figs. S1 to S6
Table S1

Note S1: Three-level model in rate equation analysis.

The coupled rate equations of the time-dependent exciton populations of the |IDE+> and |IDE-> states for $N_1(t)$ and $N_2(t)$ are as follows (*49*):

$$\frac{dN_0(t)}{dt} = -G \times N_0(t) \times (p_1 + p_2) + \Gamma_1 \times N_1(t) + \Gamma_2 \times N_2(t), \tag{S1}$$

$$\frac{dN_1(t)}{dt} = G \times N_0(t) \times p_1 \times \left(1 - \frac{N_1(t)}{n_1}\right) - \left[\Gamma_1 + \Gamma_{12} \times \left(1 - \frac{N_2(t)}{n_2}\right)\right] \times N_1(t) + \Gamma_{21} \times \left(1 - \frac{N_1(t)}{n_1}\right) \times N_2(t), \tag{S2}$$

$$\frac{dN_2(t)}{dt} = G \times N_0(t) \times p_2 \times \left(1 - \frac{N_2(t)}{n_2}\right) - \left[\Gamma_2 + \Gamma_{21} \times \left(1 - \frac{N_1(t)}{n_1}\right)\right] \times N_2(t) + \Gamma_{12} \times \left(1 - \frac{N_2(t)}{n_2}\right) \times N_1(t). \tag{S3}$$

Considering the experimental conditions of a low excitation power of 300 nW, the occupation number, N_i is much smaller than the maximum allowed number of excitons n_i for each state. Consequently, the rate equation can be simplified as follows:

$$\frac{dN_0(t)}{dt} = -G \times N_0(t) \times (p_1 + p_2) + \Gamma_1 \times N_1(t) + \Gamma_2 \times N_2(t), \tag{S4}$$

$$\frac{dN_1(t)}{dt} = G \times N_0(t) \times p_1 - (\Gamma_1 + \Gamma_{12}) \times N_1(t) + \Gamma_{21} \times N_2(t), \tag{S5}$$

$$\frac{dN_2(t)}{dt} = G \times N_0(t) \times p_2 - (\Gamma_2 + \Gamma_{21}) \times N_2(t) + \Gamma_{12} \times N_1(t). \tag{S6}$$

Time-resolved PL was conducted using sub-nanosecond laser excitation; thus, the initial population corresponds to the occupation possibility in the short time range after excitation. Under these conditions, the initial conditions of $N_0(0) = 0$, $N_1(0){:}N_2(0) = p_1{:}p_2$, and $p_1 + p_2 = 1$ were assumed. Consequently, the time-dependent exciton populations |IDE+> and |IDE-> for $N_1(t)$ and $N_2(t)$ can be described as follows:

$$\frac{dN_1(t)}{dt} = -(\Gamma_1 + \Gamma_{12}) \times N_1(t) + \Gamma_{21} \times N_2(t), \tag{S7}$$

$$\frac{dN_2(t)}{dt} = -(\Gamma_2 + \Gamma_{21}) \times N_2(t) + \Gamma_{12} \times N_1(t). \tag{S8}$$

By solving these equations, we can determine the solutions for $N_1(t)$ and $N_2(t)$ based on the assumption of the initial condition, which provides the solutions as biexponential functions as follows:

$$N_{1(2)}(t) = A_{\mathrm{f},1(2)} \exp\left(-\frac{t}{\tau_\mathrm{f}}\right) + A_{\mathrm{s},1(2)} \exp\left(-\frac{t}{\tau_\mathrm{s}}\right). \tag{S9}$$

where the τ_{f} and τ_{s} are fast and slow decay component, and $A_{\mathrm{f},1(2)}$ and $A_{\mathrm{s},1(2)}$ are the amplitudes of fast and slow decay processes in the |IDE+> and |IDE-> states, respectively. The specific expressions are as follows.

$$\tau_{\mathrm{f(s)}}{}^{-1} = \frac{1}{2}(\Gamma_1 + \Gamma_2 + \Gamma_{12} + \Gamma_{21} \pm \sqrt{(\Gamma_1 - \Gamma_2 + \Gamma_{12} - \Gamma_{21})^2 + 4\Gamma_{12}\Gamma_{21}}). \quad \text{(S10)}$$

We consider the relationship between the hybridization rate H and the transition rates $\tau_{\mathrm{f}}{}^{-1}$ and $\tau_{\mathrm{s}}{}^{-1}$. It can be simply summarized as $H = \tau_{\mathrm{f}}^{-1} - \tau_{\mathrm{s}}^{-1}$.

Moreover, we consider the relationship of amplitudes, as follows:

$$A_{\mathrm{f(s)},1} = \mp N_1(0)\frac{\Gamma_2+\Gamma_{21}-\tau_{\mathrm{f(s)}}{}^{-1}}{\tau_{\mathrm{f}}{}^{-1}-\tau_{\mathrm{s}}{}^{-1}} \pm N_2(0)\frac{\Gamma_{21}}{\tau_{\mathrm{f}}{}^{-1}-\tau_{\mathrm{s}}{}^{-1}}, \quad \text{(S11)}$$

$$A_{\mathrm{f(s)},2} = \mp N_1(0)\frac{\Gamma_{12}}{\tau_{\mathrm{f}}{}^{-1}-\tau_{\mathrm{s}}{}^{-1}} \mp N_2(0)\frac{\Gamma_1+\Gamma_{12}-\tau_{\mathrm{f(s)}}{}^{-1}}{\tau_{\mathrm{f}}{}^{-1}-\tau_{\mathrm{s}}{}^{-1}}. \quad \text{(S12)}$$

We determine the value of $N_1(0)$ and $N_2(0)$:

$$N_{1(2)}(0) = A_{\mathrm{f},1(2)} + A_{\mathrm{s},1(2)}. \quad \text{(S14)}$$

The populations of excitons in |IDE+> (N_1) and |IDE-> (N_2) can be solved under steady-state conditions: $\frac{dN_0(t)}{dt} = \frac{dN_1(t)}{dt} = \frac{dN_2(t)}{dt} = 0$ and $N_0 + N_1 + N_2 = 1$ using eq. (S4-S6) as follows(*47*, *48*):

$$N_{1(2)} = \frac{G\times(\Gamma_{2(1)}N_{1(2)}(0)+\Gamma_{21(12)})}{G\times(\Gamma_2 N_1(0)+\Gamma_{21}+\Gamma_1 N_2(0)+\Gamma_{12})+\Gamma_1\Gamma_{21}+\Gamma_2\Gamma_{12}+\Gamma_1\Gamma_2}. \quad \text{(S15)}$$

The PL intensities of the |IDE+> and |IDE-> states $I_{|\mathrm{IDE}_+>}$ and $I_{|\mathrm{IDE}_->}$can be expressed as follows:

$$I_{|\mathrm{IDE}_+>(|\mathrm{IDE}_->)} \propto \Gamma_{1(2)} \cdot \frac{G\times(\Gamma_{2(1)}N_{1(2)}(0)+\Gamma_{21(12)})}{G\times(\Gamma_2 N_1(0)+\Gamma_{21}+\Gamma_1 N_2(0)+\Gamma_{12})+\Gamma_1\Gamma_{21}+\Gamma_2\Gamma_{12}+\Gamma_1\Gamma_2}. \quad \text{(S16)}$$

The PL intensity ratio of $I_{|\mathrm{IDE}_->}$ and $I_{|\mathrm{IDE}_+>}$ defined as $I_{\mathrm{R}}(\equiv\equiv I_{|\mathrm{IDE}_->}/I_{|\mathrm{IDE}_+>})$ can be further determined using eq. (S16).

$$I_{\mathrm{R}} \equiv I_{|\mathrm{IDE}_->}/I_{|\mathrm{IDE}_+>} = \frac{\Gamma_2\times(\Gamma_1 N_2(0)+\Gamma_{12})}{\Gamma_1\times(\Gamma_2 N_1(0)+\Gamma_{21})}. \quad \text{(S17)}$$

Note S2: Photon correlation of defect-localized single-photon emission

To simultaneously demonstrate the maintenance of single-photon purity and decay dynamics of the defect-localized exciton emission under magnetic field, the photon correlation measurement is experimentally conducted in the HBT setup, represented by the second-order correlation function $g^2(\tau)$ as follows:

$$g^2(\tau) = \frac{<P_1(t_1)P_2(t_2)>}{<P_1(t_1)><P_2(t_2)>} = \frac{P_{12}}{X}, \tag{S18}$$

where $P_{1(2)}\left(t_{1(2)}\right)$ represents the detection probability at detector 1(or 2) at time $t_{1(2)}$, $< P_1(t_1)P_2(t_2) >$, denoted as P_{12} for shorthand, represents the joint probability of detecting photons events with a time delay $\tau = t_2 - t_1$. The term $< P_{1(2)}\left(t_{1(2)}\right) >$ denotes the statistical average detection possibility at detector 1(or 2), and *X* represents the product of the average detection probabilities at both detectors. This function is understood as a joint possibility of detecting a photon at time $t_1 = t$ on the start detector (detector 1) and another photon at time $t_2 = t + \tau$ on the stop detector (detector 2).

Photon correlation measurement

Photon correlation measurements of defect-localized exciton emissions are conducted in HBT setup using both continuous-wave and pulsed lasers. Under continuous-wave conditions, to account for imbalanced noise caused by dark counts, the calibrated second-order correlation function, $g^2(\tau)$, was calibrated using the expression: $g^2(\tau) = 1 - \frac{1}{\rho_1\rho_2} + \frac{C_N(\tau)}{\rho_1\rho_2}$, where ρ_1 and ρ_2 are defined as $\rho_{1(2)} = \frac{\mathrm{SBR}_{1(2)}}{\mathrm{SBR}_{1(2)}+1}$, with SBR being the signal-to-background ratio.(*52*, *54*) Under our experimental conditions, $\rho_1 = 0.9$ and $\rho_1 = 0.85$ were used for calibration.

Second-order correlation function based on rate equation

For conditions of single-photon emission under continuous-wave (cw) laser excitation, $P_1(t_1) = 1$ and considering the symmetry in the autocorrelation such that $g^2(\tau) = g^2(-\tau)$, the second-order correlation function can be mathematically expressed and simplified for $\tau > 0$. The formulation serves as a statistical representation of exciton population in the excited states as a function of time, using eq. (S19) as follows:

$$g^2(\tau) = \frac{<P_1(t_1)P_2(t_2)>}{<P_1(t_1)><P_2(t_2)>} = \frac{P(t_2)}{N_e^\infty} = \frac{N_e(t_2|N_g(t_1)=1)}{N_e^\infty}, \quad \text{(S19)}$$

where N_e and N_g indicate the populations of excitons in the excited and ground states, respectively, N_e^∞ represents the population of excitons in excited states under steady-state. Under the assumption of a three-level model, the total exciton population in the excited states is given by $N_e(t) = N_1(t) + N_2(t)$, while the exciton population in ground state is $N_g = N_0(t)$. When $N_0(0) = 1$, the second-order correlation function is simplified as follows:

$$g^2(|\tau| = t) = \frac{N_e(\tau)}{N_e^\infty} = \frac{N_1(t)+N_2(t)}{N_1+N_2}, \quad \text{(S20)}$$

where $N_1(t)$ and $N_2(t)$ represent the time-dependent populations of excitons in $|IDE_+>$ and $|IDE_->$, and the steady-state populations of excitons in $|IDE_+>$ and $|IDE_->$ are represented by N_1 and N_2. The second-order correlation function is determined using both the time-dependent ($N_{1(2)}(t)$) and the steady-state $N_{1(2)}$ exciton populations. By analyzing the time-resolved PL decay profiles of defect-localized exciton emission using the rate equation, we derived the decay rate of $\Gamma_{1(2)}$ in $|IDE_+>$ and $|IDE_->$, the transition rate of $\Gamma_{12(21)}$ between $|IDE_+>$ and $|IDE_->$ and the initial population $N_{1(2)}(0)$ that correspond to the occupation possibility $p_{1(2)}$ in $|IDE_+>$ and $|IDE_->$ as functions of external magnetic fields. From these evaluated parameters, the steady-state populations ($N_{1(2)}$) can be solved under steady-state conditions: $\frac{dN_0(t)}{dt} = \frac{dN_1(t)}{dt} = \frac{dN_2(t)}{dt} = 0$ and $N_0 + N_1 + N_2 = 1$ using eq. (S15). Meanwhile, with a given time delay, the time-dependent exciton populations ($N_{1(2)}(t)$) are determined using the rate equation in eq. (S4-6) under the initial conditions of $N_0(0) = 1$, $N_1(0) = N_2(0) = 0$, and $p_1 + p_2 = 1$.

Figure S6(A) shows the calculated results of $g^2(|\tau| = t)$ for defect-localized exciton emission (sample 1) using the derived occupation probabilities of |IDE₊> and |IDE₋> of p_1 and p_2, and the transition rates of $\Gamma_{12(21)}$ and $\Gamma_{1(2)}$ from the experimentally obtained parameters of amplitude $A_{\mathrm{f(s)}}$ and decay rates of $\tau_{\mathrm{f(s)}}^{-1}$. The black and red lines represent the cases under 0 and 1 T magnetic field, respectively.

Simulation of single-photon emission statistics under pulsed excitation

We simulated the second-correlation function of the single-photon emission statistics under pulsed excitation condition. In the simulation, the defect-localized emission under pulsed laser excitation is modeled following a double-exponential decay function using eq. (3) with the parameter of amplitude $A_{\mathrm{f(s)}}$ and decay rates of $\tau_{\mathrm{f(s)}}^{-1}$ obtained from time-resolved PL spectroscopy under magnetic field. The possibilities of detecting a photon on the start (detector 1) and stop (detector 2) detectors are considered. For each pulsed excitation period, a photon is emitted and randomly assigned to one of two-detectors simulating the experimental detection process. The probability of a photon arriving at either detector is set to 50%, ensuring an equal distribution of detection events. The total number of simulated periods is set to 250 to accumulate sufficient statistical data for the analysis. The continuous signals are converted into discrete events (0 or 1) based on the Poisson distribution method. The coincidences are recorded when both start and stop events occur (the detection values in both detectors 1 and 2 are 1) with changing the time delay within 2.5 ns time window to simulate the experimental conditions.

Figure S6(B) shows the simulation results of photon correlation for defect-localized exciton emission (sample 1) using the experimentally obtained parameters of amplitude $A_{\mathrm{f(s)}}$ and decay rates of $\tau_{\mathrm{f(s)}}^{-1}$. The insets of **Figure S6(B)** shows simulated detection (blue) and dark (purple) condition, along with the corresponding converted discrete events. The shaded area in **Figure S6(B)** shows the histograms of collected photon correlation coincidence as a function of time delay. The black and red line serve as calculated guidelines based on the symmetrical double-exponential decay function following the laser's repetition rate (10 MHz) at magnetic fields of 0 and 1 T, respectively.

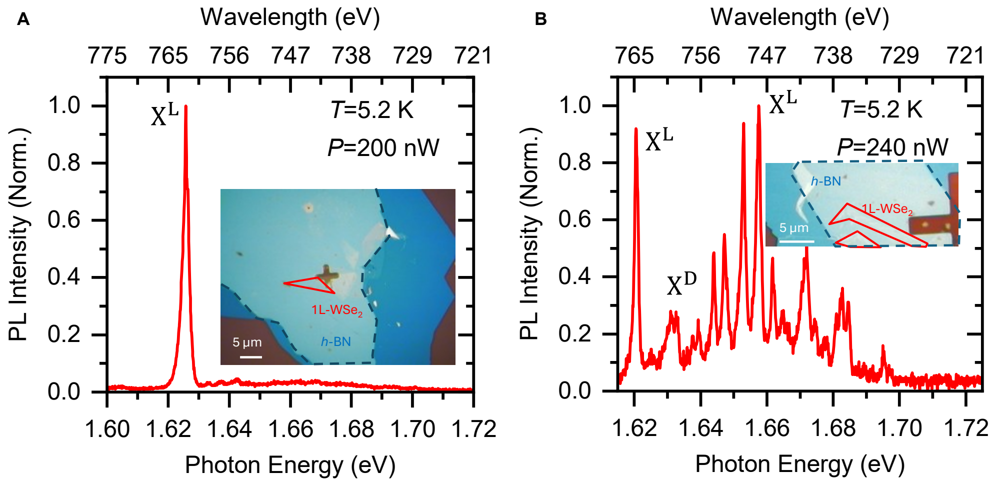

Figure S1 Low-temperature PL spectra of the defect-related emissions from different samples. (A) Low-temperature PL spectrums of the defect-related emissions from sample 2 at 5.2 K under low excitation power conditions of 200 nW. **(B)** Low-temperature PL spectrums of the defect-related emissions from sample 3 at 5.2 K under low excitation power conditions of 240 nW. The insets of (A) and (B) show the optical images of sample 2 and 3, respectively, the WSe_2 monolayer with defect sites encapsulated by top and bottom *h*-BNs. The WSe_2 monolayer and *h*-BN few layers are highlighted in red and blue dash lines, respectively. The scale bar of 5 μm is shown in the image.

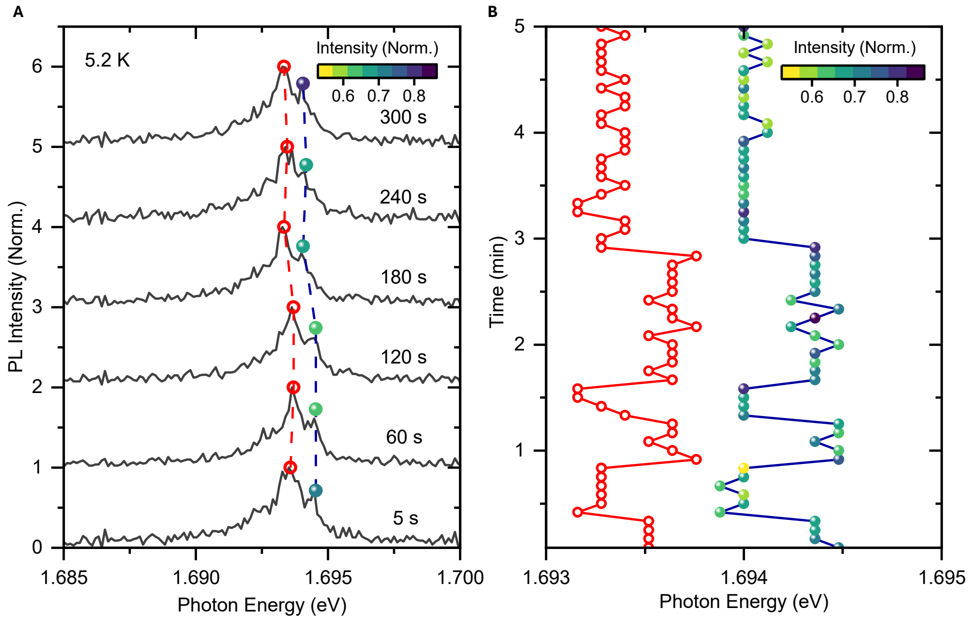

Figure S2 Time-evolution of PL spectrum from defect site emission at low temperature. (A) Time-evolution of PL spectrum from defect site emission at 5.2 K under the excitation power of 1 μW. Each PL spectrum is normalized by the lower peak intensity, and the accumulation time for each spectrum is 5 seconds. **(B)** Time-evolution of the splitting energy and the intensity of doublet at the lower (red circle) and higher energy peak (colored sphere).

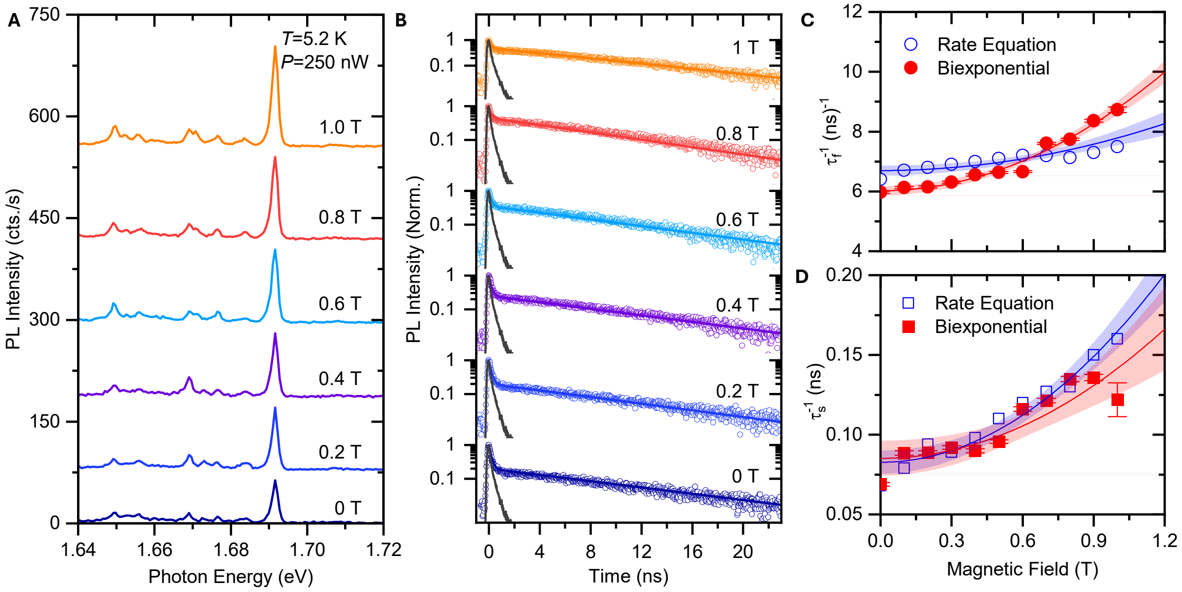

Figure S3 Magnetic field dependence of PL spectrum from defect site emission at low temperature. (A) Magnetic field dependence of PL spectra under excitation power of 250 nW with polarization-unresolved, and low spectral resolution condition. The doublet-peaks are merged in a peak at ~1.69 eV. Noted that no measurable magnetic broadening (no measurable linewidth and splitting energy changes of the doublet-peaks). **(B)** Time-resolved PL decay profiles were monitored at the photon energy of 1.69 eV. **(C)** Magnetic field dependence of the fast decay rates (τ_f^{-1}) from biexponential function (red) and derived from the rate equation (blue). **(D)** Magnetic field dependence of the slow decay rates (τ_s^{-1}) from biexponential function (red) and derived from the rate equation (blue).

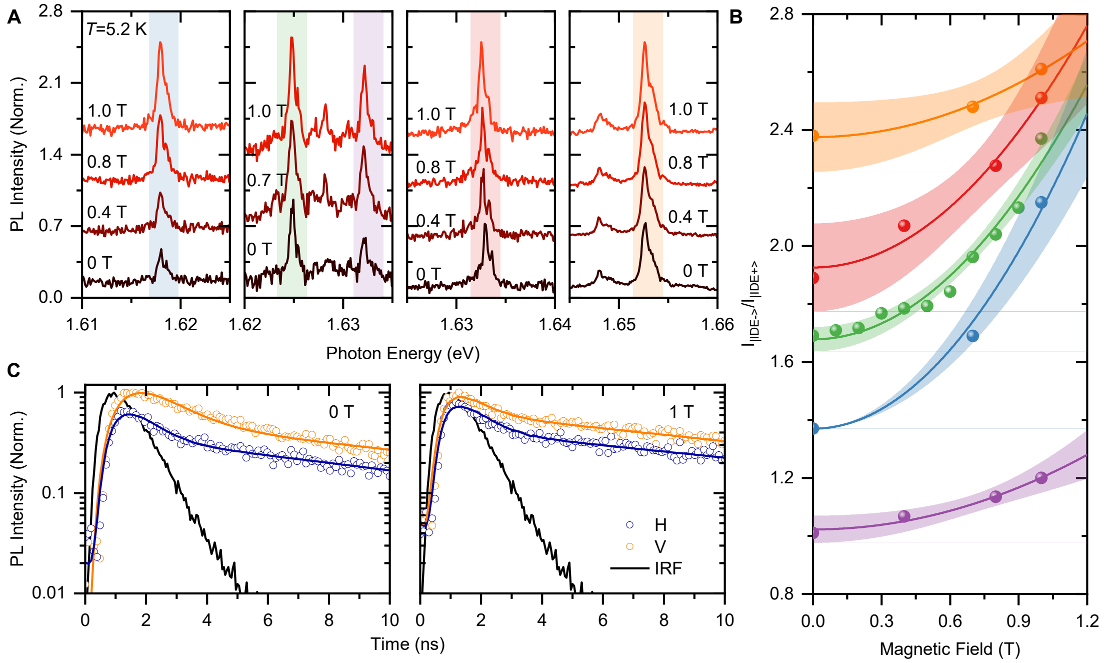

Figure S4 Magnetic field dependence of PL spectra from different positions and samples. (A) Low-temperature PL spectra of defect-localized exciton emissions with increasing the magnetic fields from different positions, and samples. **(B)** PL intensity ratio as a function of magnetic field. The color plots correspond to the data from the peaks highlighted in **Fig. S4(A)**. **(C)** Time-resolved PL decay profiles detected in H and V configurations under the magnetic field of 0 (left) and 1 T (right). The estimated errors are indicated by the colored shades.

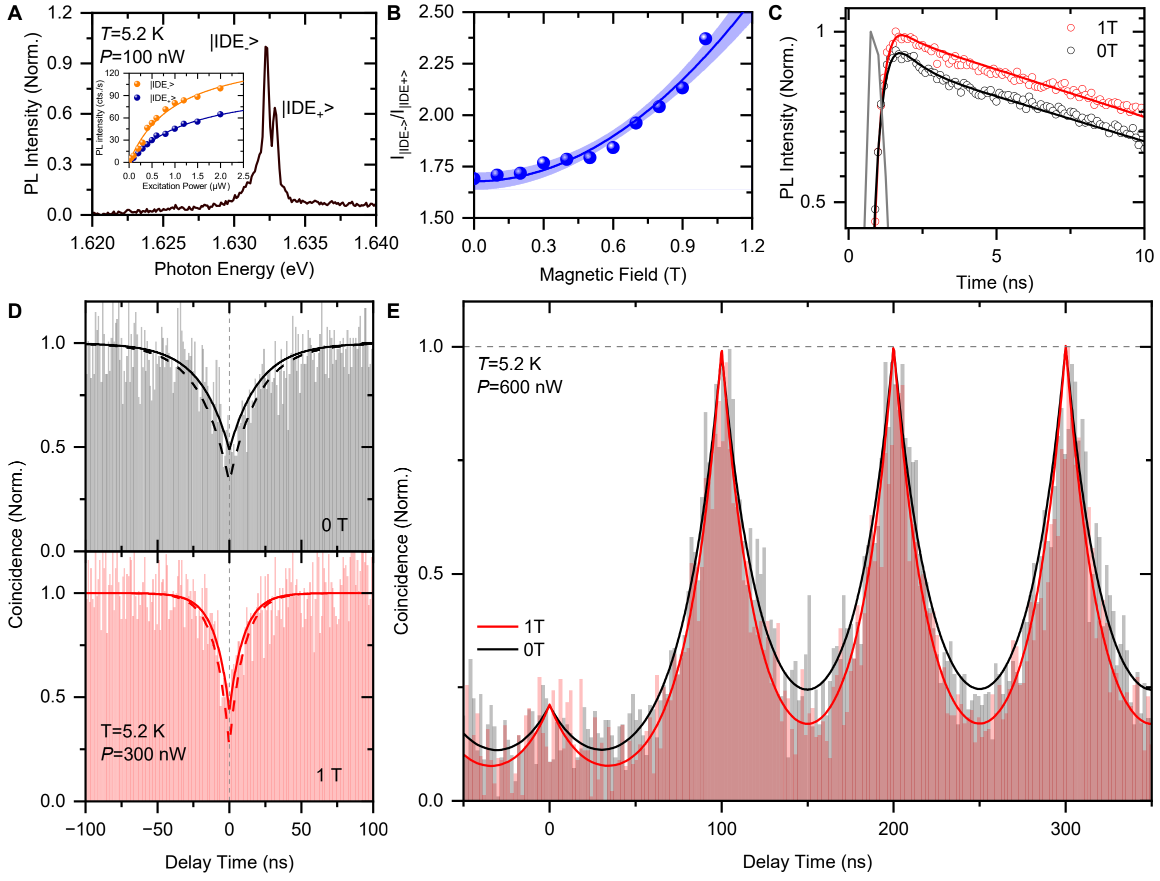

Figure S5 Low-temperature PL spectra and the magnetic brightening from monolayer WSe_2 encapsulated by *h*-BNs with defect sites on a planar Au substrate (sample 4). (A) Low-temperature PL spectra at 5.2 K under low excitation power conditions of 100 nW. The sharp peak at a photon energy of approximately 1.633 eV consists of two closely spaced doublet peaks, separated by a small energy splitting of about 0.65 meV. The inset shows the PL intensities as a function of excitation power. The results show a nonlinear saturation behavior with a saturation power P_{sat} of 1.39 and 1.04 μW for |IDE$_+$> and |IDE$_-$>, respectively. **(B)** PL intensity ratio of doublet peaks from |IDE$_-$> and |IDE$_+$> with an increasing magnetic field. The estimated errors are indicated by the colored shades. **(C)** Time-resolved PL decay profiles monitored at the photon energy of 1.633 eV. **(D)** Experimental results of photon correlation as a function of delay time from -100 ns to 100 ns using the continuous-wave laser of 300 nW at 5.2 K at 0 (upper, black) and 1 T (lower, red). The solid and dashed lines correspond to the fitting curve of $C_N(\tau)$ and calibrated $g^2(\tau)$. **(E)** Experimental results of

photon correlation under the magnetic field at 0 (black) and 1 T (red) using the pulsed laser excitation with a power of 600 nW and a repetition rate of 10 MHz. The solid lines serve as calculated guidelines based on the symmetrical double-exponential decay function following the repetition rate of laser (10 MHz).

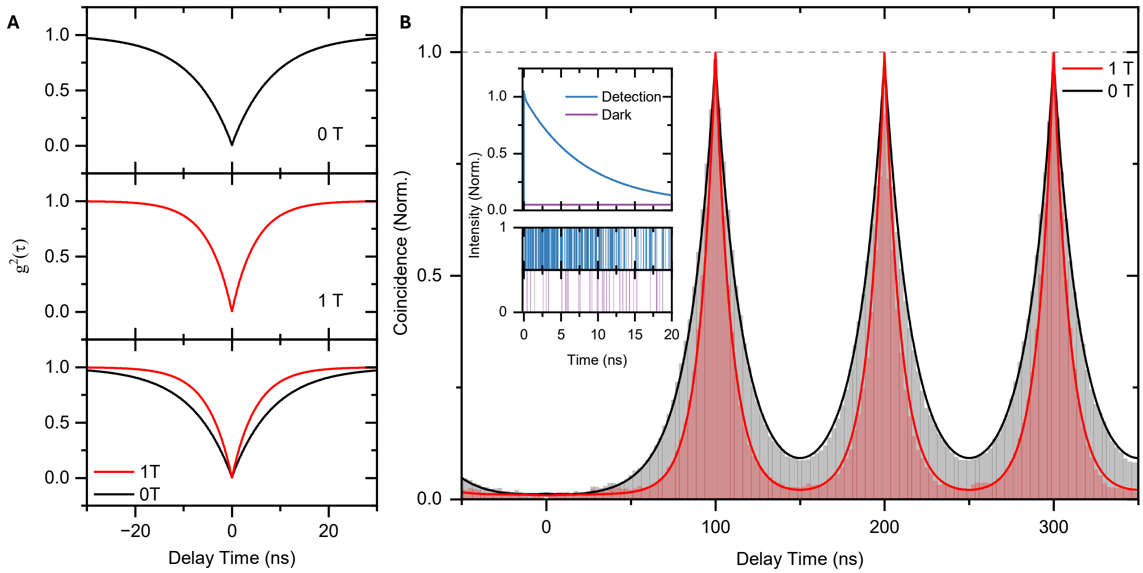

Figure S6 Calculation of second-correlation function of defect-localized exciton emission (sample 1). (A) Calculated second-order correlation function of photon emission using the rate equation analysis. **(B)** Simulation of single-photon emission statistics under pulsed excitation within a 2.5 ns time-window. The solid lines serve as calculated guidelines based on the symmetrical double-exponential decay function following the repetition rate of laser (10 MHz). Inset: Simulated decay profiles (upper) and the detected event (lower) using the parameters of time-resolved PL decay measurements. The blue and purple indicates the conditions of detection and dark in the two detectors.

Table S1: The value of the magnetic brightening factor of the dark exciton in other systems

System	Energy Splitting meV	Brightening Factor α
WSe_2	47	81×10^{-6} (*50*)
	14	96×10^{-6} (*50*)
MoS_2	98	370×10^{-6} (*50*)
WS_2	47	54×10^{-6} (*50*)
	23	39×10^{-6} (*50*)
Carbon nanotube	2.6	0.22 (*51*)
	4.5	0.14 (*51*)
